# Mobility of *β*-lactam resistance under ampicillin treatment in gut microbiota suffering from pre-disturbance

**DOI:** 10.1099/mgen.0.000713

**Published:** 2021-12-09

**Authors:** Alexander Laskey, John Devenish, Mingsong Kang, Mirjana Savic, John Chmara, Hanhong Dan, Min Lin, James Robertson, Kyrylo Bessonov, Simone Gurnik, Kira Liu, John H. E. Nash, Edward Topp, Jiewen Guan

**Affiliations:** ^1^​ Ottawa Laboratory-Fallowfield, Canadian Food Inspection Agency, Ottawa, ON, Canada; ^2^​ National Microbiology Laboratory, Public Health Agency of Canada, Guelph, ON, Canada; ^3^​ London Research and Development Centre, Agriculture and Agri-Food Canada, London, ON, Canada

**Keywords:** antibiotic resistance, plasmid transfer, gut microbiota, intestinal inflammation, antibiotic treatment

## Abstract

Ingestion of food- or waterborne antibiotic-resistant bacteria may lead to dissemination of antibiotic resistance genes (ARGs) in the gut microbiota. The gut microbiota often suffers from various disturbances. It is not clear whether and how disturbed microbiota may affect ARG mobility under antibiotic treatments. For proof of concept, in the presence or absence of streptomycin pre-treatment, mice were inoculated orally with a *β*-lactam-susceptible *Salmonella enterica* serovar Heidelberg clinical isolate (recipient) and a *β*-lactam resistant *Escherichia coli* O80:H26 isolate (donor) carrying a *bla_CMY-2_* gene on an IncI2 plasmid. Immediately following inoculation, mice were treated with or without ampicillin in drinking water for 7 days. Faeces were sampled, donor, recipient and transconjugant were enumerated, *bla_CMY-2_* abundance was determined by quantitative PCR, faecal microbial community composition was determined by 16S rRNA amplicon sequencing and cecal samples were observed histologically for evidence of inflammation. In faeces of mice that received streptomycin pre-treatment, the donor abundance remained high, and the abundance of *S*. Heidelberg transconjugant and the relative abundance of *Enterobacteriaceae* increased significantly during the ampicillin treatment. Co-blooming of the donor, transconjugant and commensal *Enterobacteriaceae* in the inflamed intestine promoted significantly (*P*<0.05) higher and possibly wider dissemination of the *bla_CMY-2_* gene in the gut microbiota of mice that received the combination of streptomycin pre-treatment and ampicillin treatment (Str–Amp) compared to the other mice. Following cessation of the ampicillin treatment, faecal shedding of *S*. Heidelberg transconjugant persisted much longer from mice in the Str–Amp group compared to the other mice. In addition, only mice in the Str–Amp group shed a commensal *E. coli* O2:H6 transconjugant, which carries three copies of the *bla_CMY-2_* gene, one on the IncI2 plasmid and two on the chromosome. The findings highlight the significance of pre-existing gut microbiota for ARG dissemination and persistence during and following antibiotic treatments of infectious diseases.

## Data Summary

All supporting data have been provided within the article or through supplementary data files. Seven supplementary figures and four supplementary tables are available with the online version of this article. The whole-genome sequencing data generated for this study can be found in NCBI BioProject PRJNA674061.

**Impact Statement**

Plasmid conjugation is an effective means for bacterial dissemination of antibiotic-resistance genes (ARGs) in the gut microbiota. Early mouse studies showed conjugative transfer of ARGs in the gut under positive antibiotic selection pressure. Recent studies demonstrated ARG transfer in the absence of antibiotic selection pressure in mice with pre-diminished gut microbiota. This study was the first to explore the impacts of interaction between antibiotic selection pressure and pre-existing gut microbiota on the dynamics of conjugative transfer of ARGs. Our findings showed that the combination, compared to either one of the two factors, positive antibiotic selection pressure and pre-existing gut dysbiosis, promoted significantly higher and possibly wider dissemination of ARGs and prolonged the persistence of ARGs in the gut microbiota. This study points to a new direction for exploring pre-existing gut microbiota for better elucidation of the mechanisms of conjugative transfer of ARGs during antibiotic treatments of infectious bacterial diseases.

## Introduction

Antimicrobial resistance (AMR; here specifically limited to antibiotics, i.e. antibacterial agents) is a serious public health issue threatening the effective prevention and treatment of an ever-increasing range of bacterial infections [1]. Long-term extensive use of antimicrobials has led to the high prevalence of antibiotic-resistant bacteria (ARB) in clinical settings, agriculture systems and the environment [2–5]. A One Health approach has been taken globally, including reduction of antimicrobial use in human medicine and agriculture, in order to reduce AMR transmission to humans via the environment and food consumption [6–9]. To evaluate the potential health impacts of exposure to food- or waterborne contamination with ARB, it is critical to understand the dynamics of ingested ARB and the antibiotic-resistance genes (ARGs) that they carry in the host gut microbiome, and how this varies according to factors such as treatment with antibiotics.

*Firmicutes* and *Bacteroidetes* are the dominant phyla in a healthy gut microbiota [10]. These bacteria produce short-chain fatty acids that maintain a mildly acidic gut environment against the colonization of opportunistic *Enterobacteriaceae* [11, 12]. Members of the gut microbiota also compete with the intruding bacteria for niches and nutrients [13], and educate and maintain the host immune system to generate rapid and efficient response against intruders [14, 15]. Thus, a healthy gut microbiota may resist the colonization of ARB following ingestion and therefore reduce the opportunity for horizontal transfer of plasmid-borne ARGs.

Plasmid conjugation is an efficient means for dissemination of ARGs from ARB to commensal and pathogen bacteria in the gut microbiota [16, 17]. In general, antibiotics cause dysbiosis, thereby enabling ARB colonization and promoting conjugative ARG dissemination in the gut microbiome [18]. However, the results from modelling and *in vitro* experiments [19] show that antibiotics have the potential to promote or suppress conjugation through selection or inhibition of the donor, recipient or transconjugant. In the pre-existing normal mouse gut microbiota, antibiotics also have selective and suppressive effects on donor and recipient that enhance or inhibit conjugation of ARG-bearing plasmids during antibiotic treatments [20]. Since the gut microbiota often suffers from disturbance by various factors, such as diet, bacterial infection and antibiotic treatment, it is important to understand the effects of antibiotics on conjugative transfer of ARGs in microbiota with pre-existing disturbance. As a proof of concept study, we explored the impact of ampicillin on mobility of *β*-lactam resistance in the gut microbiota that had suffered pre-disturbance by streptomycin.

In a previous study, co-infection of mice with multiple *β*-lactam-resistant bacterial strains favoured colonization of *Escherichia coli* O80:H26 and enabled conjugative transfer of its *bla_CMY-2_* gene via an IncI2 plasmid under ampicillin treatment [21]. Building on this model, we used the *E. coli* O80:H26 strain as a donor, along with a *β*-lactam-susceptible *Salmonella* Heidelberg, as a recipient in the present study. To elucidate how antibiotics may influence conjugation in the gut microbiota, we compared the dynamics of the donor, recipient, transconjugant and *β*-lactam resistance genes in mice that received ampicillin treatment, streptomycin pre-treatment, a combination of streptomycin pre-treatment and ampicillin treatment, or no antibiotics as control.

## Methods

### Bacteria

*E. coli* O80:H26 (EC-107) is a multi-antibiotic-resistant strain isolated from a chicken farm in Ontario, Canada. *E. coli* O80:H26 carries five plasmids: IncI2, IncY, IncFII, ColRNAI and a plasmid with no detectable Inc type [21]. IncI2 is a conjugative plasmid encoding a *bla_CMY-2_* gene and IncY is a mobilizable plasmid encoding a *bla_TEM-1B_* gene. *In vitro* and *in vivo* transfer of the IncI2 but not IncY plasmid was detected using *E. coli* O80:H26 as donor and *E. coli* O16:H48 as recipient in the previous study [21].

*S. enterica* serotype Heidelberg (12–6342) is a human clinical isolate that does not carry any *bla* gene and is susceptible to *β*-lactam antibiotics [22]. *S*. Heidelberg carries two plasmids: IncX1 and ColRNAI. In the present study, *S*. Heidelberg was used as a recipient of *β*-lactam resistance. To facilitate recovery of *S*. Heidelberg, a spontaneous rifampicin-resistant mutant was generated. In brief, *S*. Heidelberg was cultured overnight in Luria–Bertani (LB; Miller formulation, Difco, Fisher Scientific, Ottawa, ON, Canada) broth at 37 °C. A 1.0 ml overnight culture was pelleted, resuspended in 100 µl LB broth and spread on LB agar supplemented with 50 µg ml^−1^ rifampicin (LB-R). After 24 h incubation, resistant colonies were selected and sub-cultured on LB-R agar 20 times to generate and maintain a *S*. Heidelberg rifampicin-resistant mutant culture.

### *In vitro* conjugation

*In vitro* conjugation between *E. coli* O80:H26 (donor) and *S*. Heidelberg (recipient) was assessed in 10^−1^ × LB broth as described by Laskey *et al*. [21]. Enumeration of the donor, recipient and transconjugant bacteria was performed using Chromocult agar (EMD Millipore, Toronto, ON, Canada) supplemented with 4 µg ml^−1^ cefotaxime (CHR-F), XLT4 agar (Difco) with 50 ug ml^−1^ rifampicin (XLT4-R) and XLT4 agar with 4 µg ml^−1^ cefotaxime and 50 µg ml^−1^ rifampicin (XLT4-FR), respectively. Conjugation frequency is expressed as the ratio of transconjugant to donor enumerated at the end of the mating incubation.

### *In vivo* conjugation

**Fig. 1.**
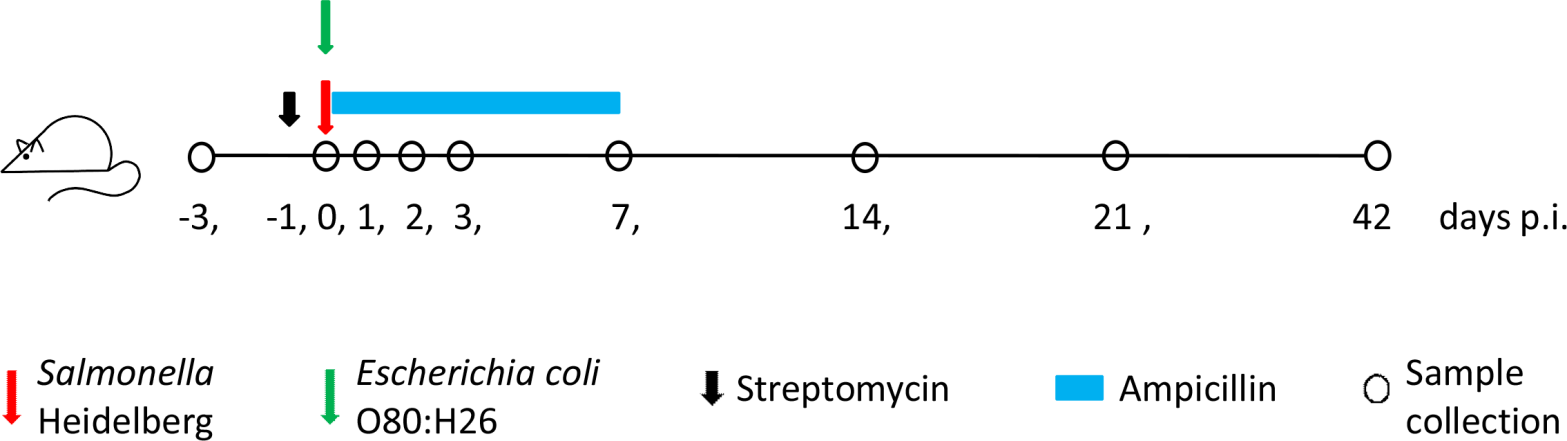
Schedule of procedures for mice that were inoculated with the *Salmonella* Heidelberg recipient and the *Escherichia coli* O80:H26 donor, and provided with treatments of streptomycin followed by ampicillin (SH-EC/Str–Amp). Sample collection was performed on various days post-infection (p.i.), as indicated.

Experiments and procedures involving mice conformed to guidelines established by the Animal Care Committee at the Ottawa Laboratory-Fallowfield, Canadian Food Inspection Agency. Female C57BL/6 mice at the age of 28 days were purchased from Charles River Laboratories (Saint Constant, QC, Canada). Mice were mixed and acclimatized for 2 weeks prior to bacterial inoculation or antibiotic treatment, and then housed three or four per cage (Optimice, Animal Care Systems, CO, USA) with water and feed was provided *ad libitum*. A total of 68 mice were randomly assigned into 2 sets of 4 groups (a total of 8 groups) to investigate the shedding of the donor and/or recipient bacteria and the transfer of plasmids carrying *β*-lactam resistance genes under various antibiotic treatments (Table 1). One set of mice were inoculated with only the recipient bacteria and the other set were inoculated with the recipient followed by the donor bacteria 1 h later. Bacterial inocula (100 µl) prepared from log-phase culture containing ~3.0×10^8^ colony-forming units (c.f.u.) of the recipient or donor bacteria in buffered peptone water (Difco) were administrated via oral gavage. Four different treatments were tested in this study: (1) ampicillin treatment (Amp), provided immediately following bacterial inoculation via drinking water (0.16 mg ml^−1^, equivalent to 30 mg ampicillin kg^−1^ of body weight per day) *ad libitum* for 7 days; (2) streptomycin pre-treatment (Str), provided once via oral gavage (20 mg per mouse) 24 h before bacterial inoculation; (3) a combination of streptomycin pre-treatment and ampicillin treatment (Str–Amp); and (4) a control without the use of antibiotics (Ctl). Each treatment was applied to a group of mice from each set, one with only the recipient inoculation and the other with both the recipient and the donor inoculation. Fig. 1 shows the schedule of procedures for the group of mice which received inoculation of both *S*. Heidelberg and *E. coli* O80:H26, and treatments with both streptomycin and ampicilln. Faecal pellets were collected from all mice on −3 (baseline), 0 (bacterial inoculation), 1, 2, 3, 7, 14, 21 and 42 day post-infection (p.i.). Pellets were processed as described by Laskey *et al.* [21] for DNA extraction and bacterial enumeration. The three selective agars, CHR-F, XLT4-R and XLT4-FR, were used to enumerate the donor, recipient and putative transconjugant bacteria, respectively, with a detection limit of 2.2 log_10_ c.f.u. g^−1^ in faeces. At 7 days p.i., some of the mice inoculated with both recipient and donor were euthanized, consisting of five, five, six and four mice from the groups with the Amp, Str and Str–Amp treatment and the control, respectively. Tissue specimens of the cecum were collected from these mice and immediately stored in 10 % neutral buffered formalin for histological examinations.

**Table 1.** Treatment groups in mouse experiments

Group	Donor	Recipient	Treatment*	*n*†
SH/Ctl	No	*S.* Heidelberg	Control (no antibiotic)	6
SH/Amp	No	*S.* Heidelberg	Ampicillin	6
SH/Str	No	*S.* Heidelberg	Streptomycin	6
SH/Str–Amp	No	*S.* Heidelberg	Streptomycin followed by ampicillin	6
SH-EC/Ctl	* E. coli* O80:H26	*S.* Heidelberg	Control (no antibiotic)	10 (4)
SH-EC/Amp	* E. coli* O80:H26	*S.* Heidelberg	Ampicillin	11 (5)
SH-EC/Str	* E. coli* O80:H26	*S.* Heidelberg	Streptomycin	11 (5)
SH-EC/Str–Amp	* E. coli* O80:H26	*S.* Heidelberg	Streptomycin followed by ampicillin	12 (6)

*Ampicillin treatment was provided via drinking water (0.16 mg ml^−1^) immediately following bacterial inoculation for 7 days, streptomycin treatment was provided via oral gavage (20 mg per mouse) once 24 h before bacterial inoculation, streptomycin followed by ampicillin treatment was the sequential combination of streptomycin and ampicillin treatment.

†*n* is the number of mice used in the experiment, and the number in parentheses represents the number of mice that were euthanized on 7 day post-infection for collection of cecum tissues for histological analysis.

Amp, Ampicilin; Ctl, control; EC, *Escherichia coli* O80:H26; SH, *Salmonella* Heidelberg; Str, streptomycin.

### Whole genome sequencing

Putative transconjugant bacteria were whole-genome sequenced and sequence data were analysed using the MOB-suite software tool v2.1.0 [23, 24]. Representative putative transconjugant colonies isolated from selective agar plates (up to five colonies per time point) were subjected to genomic DNA extraction as described by Laskey *et al.* [21]. Whole-genome sequencing was performed using an Illumina MiSeq system and/or an Oxford Nanopore MinION sequencer (Oxford Nanopore, Cambridge, MA, USA) at the National Microbiology Laboratory (Guelph, ON, Canada). All short- and long-read data were deposited to the National Center for Biotechnology Information (NCBI) under BioProject PRJNA674061. The raw reads along with assemblies of genomes and plasmids were deposited under the BioSample accession numbers (SAMNs) listed in Table S1 (available in the online version of this article). Illumina raw reads were assembled using the shovill v1.1.0 pipeline (https://github.com/tseemann/shovill) with the following parameters: --gsize 5000000 --assembler spades --trim --depth 0 --mincov 0 --minlen 0. Hybrid assemblies utilizing Nanopore and Illumina raw reads were assembled using unicycler v0.4.7 run under default parameters. All assemblies were manually reviewed to confirm the completeness of the chromosome and any plasmids present. As part of the validation process, complete plasmid assemblies were mapped against raw reads using the Snippy [25] pipeline to assess coverage and any potential coverage gaps. The assembled sequences were further analysed using the MOB-suite v2.1.0. and Prokka [26] software tools. An IncI1 plasmid map was rendered using the UGENE software [27] and the plasmid was annotated using Prokka v1.13.3. A gene map in a chromosomal range of an *E. coli* O2:H6 transconjugant was rendered using the DNA Features Viewer Python library (https://github.com/Edinburgh-Genome-Foundry/DnaFeaturesViewer) and the partial genome was annotated using Prokka v1.13.3.

### 16S rRNA gene amplicon sequencing

DNA extracted from mouse faecal pellets was subjected to 16S rRNA gene amplicon sequencing as described by Laskey *et al.* [21] at the Ottawa Laboratory-Fallowfield, Canadian Food Inspection Agency (Ottawa, ON, Canada). In brief, the V3–V4 region of the 16S ribosomal RNA gene was amplified through PCR [28]. Libraries were prepared and sequenced using a MiSeq system (Illumina). Raw read data was demultiplexed and then analysed using Qiime2 [29] through a modified version of the Qiime2 pipeline created by Forrest Dusseault (https://github.com/forestdussault/AmpliconPipeline). Data analysis and visualization were performed using the R package and GraphPad Prism 8.0 software (San Diego, CA, USA).

### Quantitation of *β*-lactam resistance genes

The abundance of *bla_CMY-2_*, *bla_TEM-1_* and 16S rRNA genes in mouse faecal pellets was determined by qPCR [30–32, Table S2]. Genomic DNA extracted from an overnight culture of *E. coli* O80:H26 with the DNeasy Blood and Tissue kit (Qiagen, Toronto, ON, Canada) was used for the generation of standard curves. Purified *E. coli* O80:H26 DNA was quantified and diluted in nuclease-free water to serial 10-fold concentrations from 4 fg µl^−1^ to 4 ng µl^−1^ to provide a 6 log_10_ range of quantitation. DNA extracted from mouse faecal pellets was diluted to 0.4 ng µl^−1^ for quantitation of the *bla_CMY-2_* and *bla_TEM-1_* genes and to 0.4 pg µl^−1^ for the 16S rRNA gene. All qPCRs were performed using a QuantStudio 3 Real-Time PCR System (Thermo Fisher, Nepean, ON, Canada). Reaction mixture (25 µl) contained 12.5 µl Power SYBR Green PCR master mix (Thermo Fisher), 1.0 µl forward and 1.0 µl reverse primers (final concentrations of 0.3 µM for *bla_CMY-2_*, 0.2 µM for *bla_TEM-1_* and 0.3 µM for 16S rRNA; primer sequences as shown in Table S2), 0.2 µl (0.2 U) Antarctic Thermolabile UDG (New England Biolabs, Whitby, ON, Canada), 5 µl template DNA and 6.3 µl nuclease-free water. The PCR programme included an initial incubation at 95 °C for 10 min followed by 40 cycles of denaturation at 95 °C for 15 s, annealing and elongation at the given temperatures (Table S2) for 30 s. A final stage with temperature ramping from 65–95 °C was included for analysis of the melting curves of PCR products to confirm the specificity of PCRs. Duplicate wells in duplicate PCR runs were performed for each DNA sample.

### Histology analysis

Cecum tissue specimens were prepared in Swiss rolls and fixed in 10 % (v/v) neutral buffered formalin for at least 24 h. Fixed tissues were embedded in paraffin, sectioned and stained with haematoxylin and eosin [33]. Lesions were evaluated qualitatively and assigned a score based on intensity of infiltrate and inflammation, ulceration and necrosis observed in the most severely affected area of the section. Scores, adapted from methods described by Erben *et al.* [34], were assigned as follows: 0, normal; 1, minimal; 2, mild; 3, moderate; and 4, marked. The following factors were taken into consideration for scoring: (a) leucocyte density and location: none, 0; minimal mucosal/submucosal, 1; mild mucosal/submucosal, 2; mild to moderate mucosal/submucosal, 3; moderate to marked transmural, 4; (b) goblet cell loss: none, 0; minimal, 1–2; (c) mucosal architecture change related to necrosis (crypt hyperplasia, erosion/ulceration, crypt abscess): none, 0; minimal, 1; minimal/mild, 2; mild, 3; moderate/marked, 4.

### Statistical analysis

Differences in the conjugation frequency, mean abundance of each target bacterium and relative abundance of each phylum or family in the 16S rRNA gene community profiles between the treatment groups on the same sampling day were analysed with Brown–Forsythe and Welch analysis of variance (ANOVA) tests. Differences in the inflammatory score between the treatment groups were analysed using the Kruskal–Wallis test. All correlations were tested using the Pearson correlation test. The treatment groups contained up to 12 mice (Table 1), and a mean value derived from technical replicates from one faecal pellet of each mouse on each sampling date represents one datum point. Data were analysed using GraphPad Prism 8.0 software. A *P* value <0.05 was considered statistically significant.

## Results

### *In vitro* and *in vivo* conjugation

**Fig. 2.**
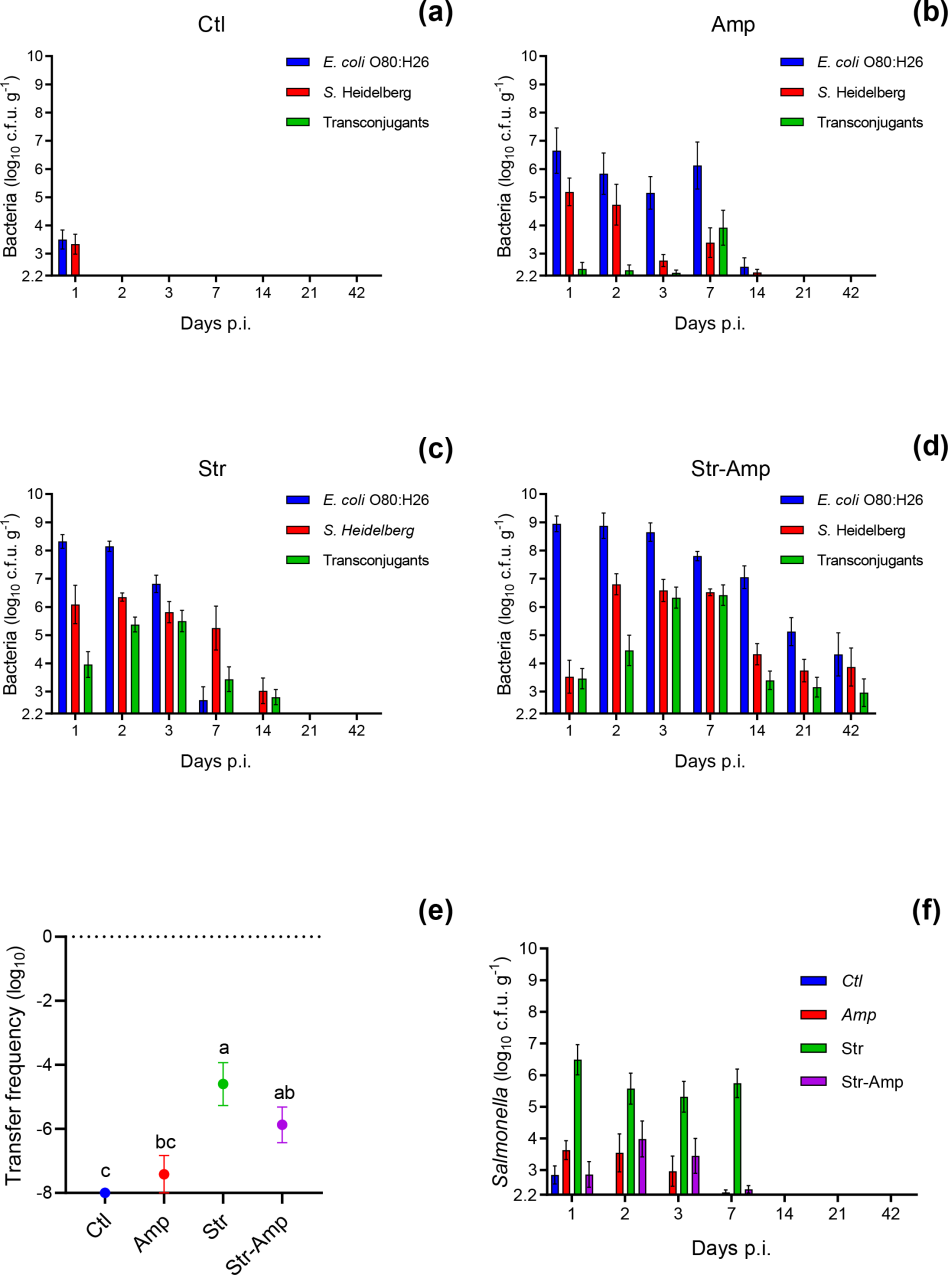
Enumeration of *Escherichia coli* O80:H26, *Salmonella* Heidelberg and the *S.* Heidelberg transconjugant (mean±se) in faecal samples from mice that received both the recipient and donor inoculation and the treatment of (a) no antibiotic (Ctl), (**b**) ampicillin (Amp), (**c**) streptomycin (Str) or (d) streptomycin followed by ampicillin (Str–Amp); *n*=10, 11, 11 and 12 for (a–d), respectively, by 7 days p.i., and *n*=6 per treatment group thereafter. (**e**) Conjugation frequency (mean±se) at 1 day p.i. is expressed as the ratio of transconjugant to donor, and mean values labelled without common letters are of significant (*P*<0.05) difference based on Brown–Forsythe and Welch’s ANOVA tests. (**f**) Enumeration of *S.* Heidelberg (mean±se) in faecal samples from mice that only received the recipient inoculation and the treatment of Ctl, Amp, Str or Str–Amp; *n*=6 per treatment group.

*β*-lactam resistance was transferable from the *E. coli* O80:H26 donor to the *S.* Heidelberg recipient *in vitro* and *in vivo* based on enumeration of the donor, recipient and putative transconjugant according to their phenotypes. The *in vitro* conjugation frequency was 1.5×10^−5^ (data not shown). The *in vivo* frequency at 1 day p.i. was 3.9×10^−8^, 2.5×10^−5^ and 1.3×10^−6^ of the Amp, Str and Str–Amp groups, respectively (Fig. 2e). Conjugation was not detected at a limit of 1.0×10^−8^ in the control group. The frequency of the Str and Str–Amp groups was significantly [F^*^(3, 32.14)=8.201, *P*=0.0003; W(3, 18.33)=12.83, *P*<0.0001] higher than that of the control group. Although the donor abundance in the Str–Amp group was the highest among all groups at 1 day p.i., the transfer frequency of the Str–Amp group was lower than that of the Str group, likely due to limited recipient abundance (Figs 2a-e and S1a). However, from 3 to 42 days p.i., the abundance of donor, *S.* Heidelberg and *S.* Heidelberg transconjugant in the Str–Amp group was the highest among all groups (Figs 2a–d and S1). In addition, *S*. Heidelberg was shed in faeces for a longer period of time following co-infection with the donor and recipient than mono-infection with the recipient under all antibiotic treatments (Fig. 2b–d and f). Furthermore, the abundance of *S*. Heidelberg was significantly higher in faeces of mice co-infected with the donor and recipient than those mono-infected with the recipient from 2 to 42 days p.i. under Str–Amp treatment (Figs 2d, f and S2d).

### Horizontal transfer of conjugative plasmids

**Fig. 3.**
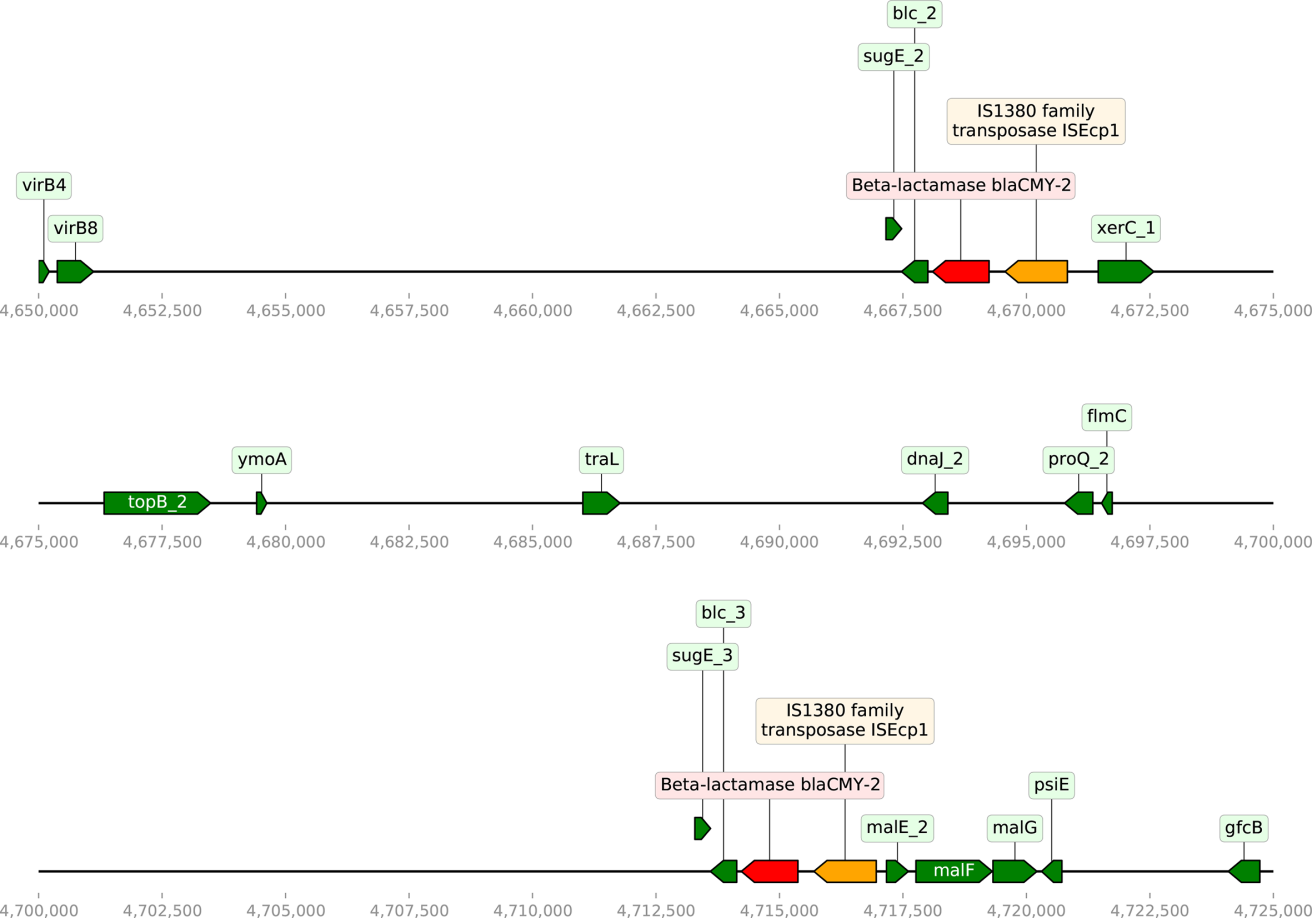
Gene map of the *Escherichia coli* O2:H6 (SAMN16634233) genome in the 4 650 000–4 750 000 bp range showing the two copies of the *bla_CMY2_* (red) gene next to IS1380 family transposase ISEcp1 (yellow). The following genes were identified in the vicinity of the *bla_CMY2_* gene: s*ugE*, quaternary ammonium compound resistance protein; *blc*, outer-membrane lipoprotein; *xerC*, tyrosine recombinase; *malE*, maltose-binding periplasmic protein; *malF*, maltose transport system permease protein; *malG*, maltose transport system permease protein; *psiE*, phosphate starvation-inducible membrane protein.

To confirm horizontal transfer of *β*-lactam resistance, putative transconjugants were subjected to whole-genome sequencing analysis and plasmid characterization with the MOB-suite tool v2.1.0. Sequencing information on the representative transconjugants is available in NCBI BioProject PRJNA674061. According to sequence data analysis, three different transconjugant strains were recovered: (1) *S*. Heidelberg carrying an IncI2 plasmid [SH-(IncI2)], (2) *S*. Heidelberg carrying both an IncI1 and an IncI2 plasmids [SH-(IncI1, IncI2)] and (3) a mouse commensal *E. coli* O2:H6 strain carrying both an IncI1 and an IncI2 plasmids [EC-(IncI1, IncI2)]. EC-(IncI1, IncI2) appeared a creamy white colour on XLT4-FR agar plates used for selective culturing *S.* Heidelberg transconjugant. Only SH-(IncI2) was recovered in the Amp group, both SH-(IncI2) and SH-(IncI1, IncI2) recovered in the Str group and all three transconjugant strains recovered in the Str–Amp group (Tables 2 and S1). The IncI1 or IncI2 plasmids in different bacterial strains shared identical size and MOB-suite plasmid cluster code, suggesting plasmid transfer between different bacterial hosts. Specifically, the IncI2 plasmid was transferred from the *E. coli* O80:H26 donor to the *S.* Heidelberg recipient, and from either the *E. coli* O80:H26 donor or the *S.* Heidelberg transconjugant to *E. coli* O2:H6. The IncI1 plasmid was possibly transferred from *E. coli* O2:H6 or other bacteria in the gut microbiota to the *S*. Heidelberg transconjugant, as neither the *E. coli* O80:H26 donor nor the *S.* Heidelberg recipient carries the IncI1 plasmid. The IncI1 conjugative plasmid belongs to cluster 476 (MOB-suite v2.1.0) and contains no ARGs (Fig. S3). Most of the identified genes on the IncI1 plasmid were of *E. coli* origin, suggesting a stable long-lived plasmid residence in *E. coli *. These genes are related to stress response, such as SOS response, toxin–antitoxin system and plasmid mobility, and could possibly contribute to conjugative transfer of the IncI1 plasmid. Analysis of the *E.coli* O2:H6 complete genome (NCBI BioSample SAMN16634233) identified two copies of the *bla_CMY-2_* gene on the chromosome and one copy on the IncI2 plasmid. Both copies of the *bla_CMY-2_* gene on the chromosome are adjacent to transposase ISEcp1 (Fig. 3), suggesting a possible movement of the *bla_CMY-2_* gene from the IncI2 plasmid to the chromosome.

**Table 2.** Transconjugant isolated from mice under different antibiotic treatments

Treatment*	Transconjugant† (plasmid)	Isolation day	Accession representative‡
Ctl	nd		
Amp	SH-(IncI2)	7 days p.i.	SAMN16634224
Str	SH-(IncI2)	1, 2, 3, 7 days p.i.	SAMN16634202
	SH-(IncI1, IncI2)	3, 7 days p.i.	SAMN16634220
Str–Amp	SH-(IncI2)	1, 2, 3 days p.i.	SAMN16634250
	SH-(IncI1, IncI2)	7, 14, 21, 42 days p.i.	SAMN16634227
	EC-(IncI1, IncI2)	14, 42 days p.i.	SAMN16634233

*Mice received inoculation of the *Salmonella* Heidelberg recipient and then the *Escherichia coli* O80:H26 donor bacteria and treatment of no antibiotic (Ctl), ampicillin (Amp), streptomycin (Str) or streptomycin followed by ampicillin (Str–Amp).

†nd, not detected; SH=*Salmonella* Heidelberg; EC=*Escherichia* coli O2:H6.

‡BioSample accession number of the representative isolate, each includes chromosomal and plasmid components of a single isolate.

### Dynamics of the *β*-lactam resistance genes

**Fig. 4.**
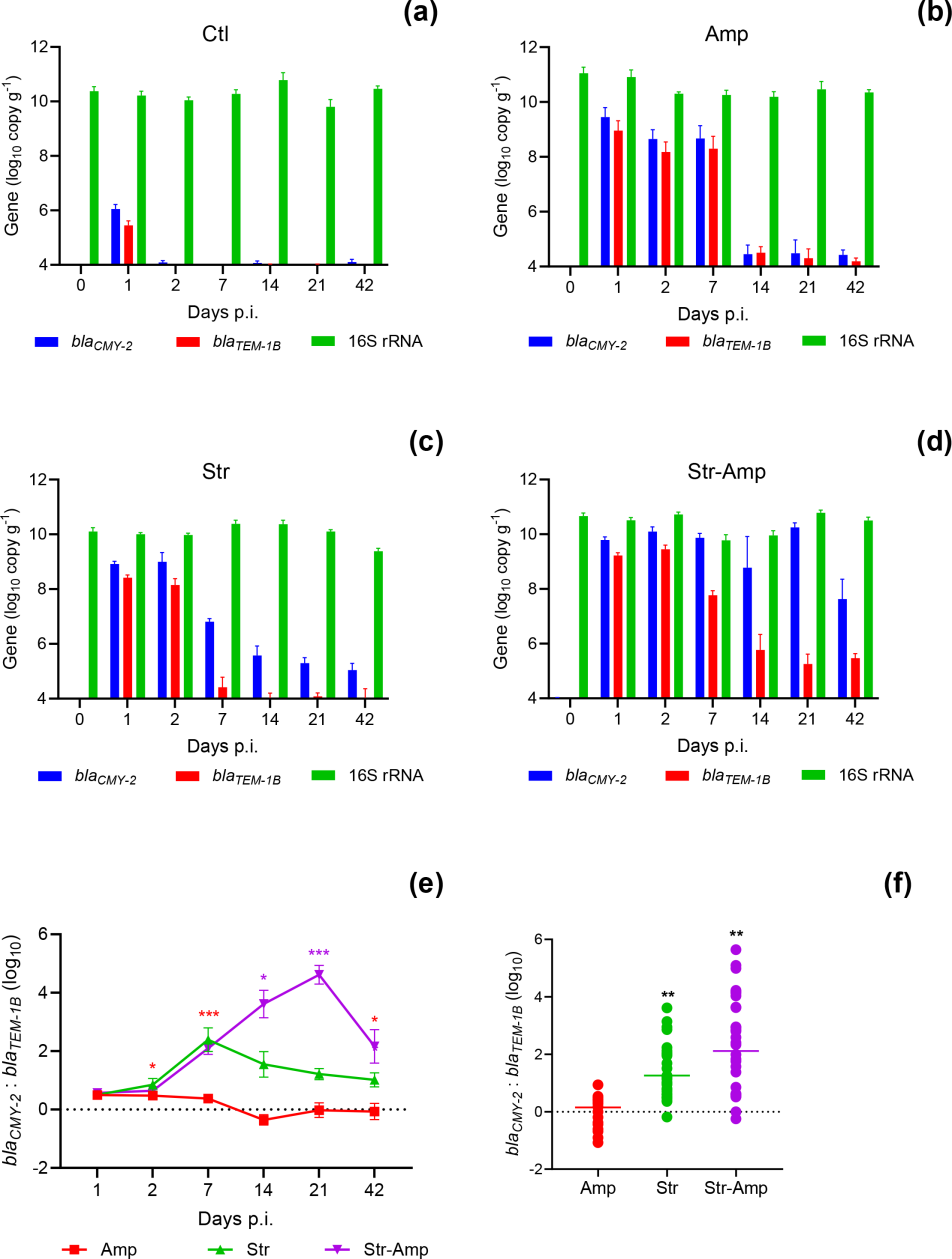
Quantitation of the *bla_CMY-2_*, *bla_TEM-1B_* and 16 s rRNA genes (mean+se) in mouse faecal samples. Mice received inoculation of *Salmonella* Heidelberg and then *Escherichia coli* O80:H26 and treatment of (a) no antibiotic (Ctl), (**b**) ampicillin (Amp), (**c**) streptomycin (Str) or (d) streptomycin followed by ampicillin (Str–Amp); *n*=6 per group. The mean ratio of the *bla_CMY-2_* to *bla_TEM-1B_* gene on each sampling day (e) or individual ratio of all samples (f) from each antibiotic treatment group. In (e), purple stars represent significant difference (*P<0.05, **P<0.01, ***P<0.001) in the mean ratio between the Str–Amp and any other treatment groups and red stars between the Str–Amp and Amp groups. In (f), the stars represent significant difference between a treatment group and any other treatment groups based on Brown–Forsythe and Welch’s ANOVA tests.

The *E. coli* O80:H26 donor carries one copy of the IncI2 plasmid encoding one copy of the *bla_CMY-2_* gene and one copy of the IncY plasmid encoding one copy of the *bla_TEM-1B_* gene. The abundance of both genes was determined by qPCRs for investigating their transmission dynamics. Neither of the two genes was detected at a detection limit of 4.0 log_10_ copies g^−1^ of faeces from mice mono-infected with the *S*. Heidelberg recipient (data not shown). From mice co-infected with the donor and recipient, both genes were detected for only 1 day in the control group, as the donor bacteria passed transiently through the mouse gut (Fig. 4a). In antibiotic treatment groups, the dynamics of *bla_TEM-1B_* abundance agreed with *E. coli* O80:H26 shedding patterns (Figs 4b–d and 2b–d). The abundance of *bla_TEM-1B_* and *E. coli* O80:H26 are positively correlated (Pearson’s correlation coefficient *r=0.83*, *P*<0.001, Fig. S4a), suggesting clonal transmission of *bla_TEM-1B_* along with the donor. In comparison, the abundance of *bla_CMY-2_* and *E. coli* O80:H26 are negligibly correlated (Pearson’s correlation coefficient *r=0.19*, *P*<0.001, Fig. S4b). The dynamics of *bla_CMY-2_* and *bla_TEM-1B_* were similar in the Amp group (Fig. 4b). In comparison, *bla_CMY-2_* abundance remained high while *bla_TEM-1B_* abundance decreased in the Str–Amp group after cessation of the ampicillin treatment (Fig. 4d). The ratio of *bla_CMY-2_* to *bla_TEM-1B_* was significantly (*P*<0.05) higher from 2 to 42 days p.i. in the Str–Amp group compared to the Amp group (Fig. 4e, f).

### Correlations between gut microbiota and transmission of *β*-lactam resistance genes

**Fig. 5.**
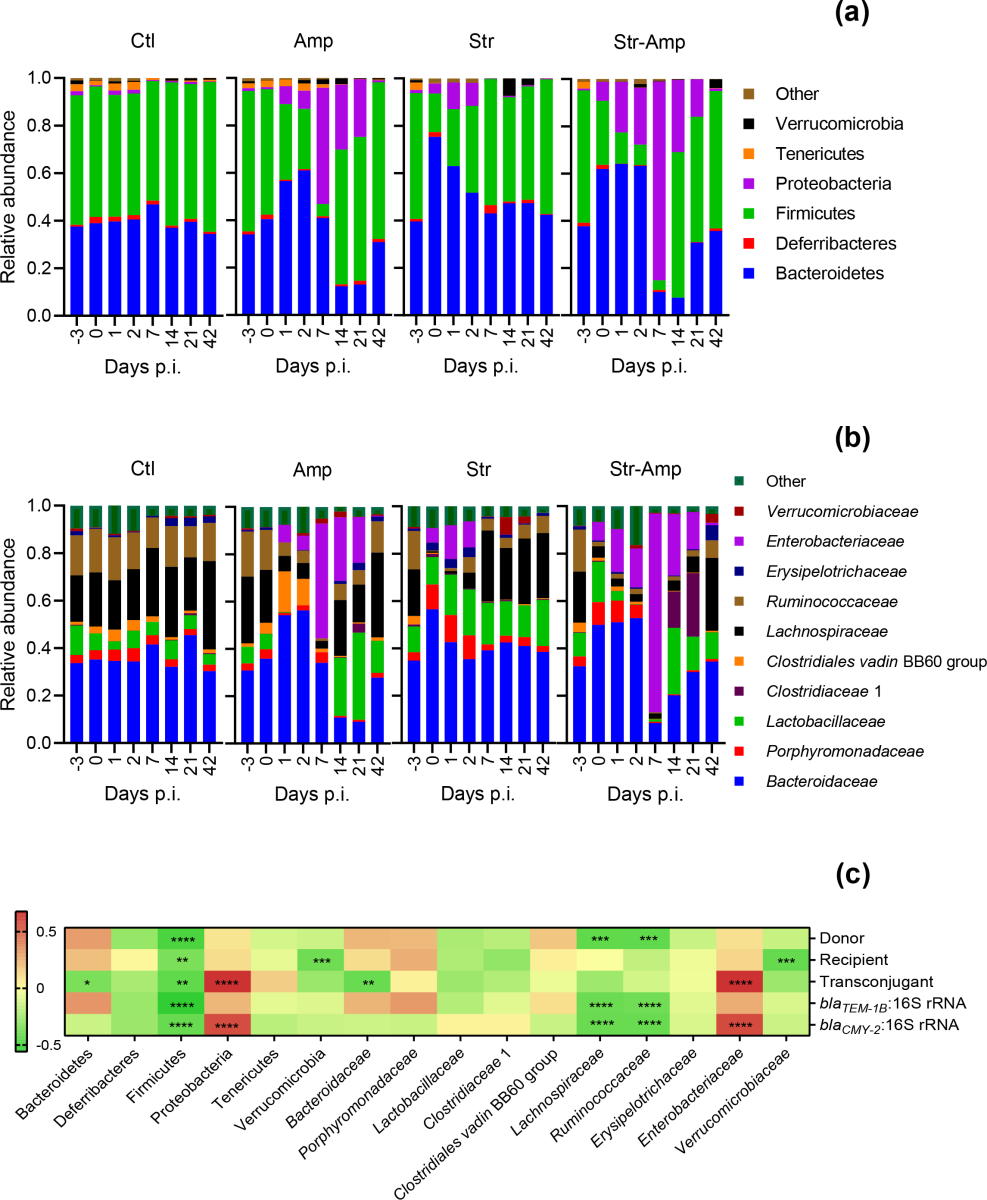
Microbial community composition shown as mean relative abundance of (a) phyla and (b) families based on sequencing of the 16S rRNA gene from mouse faecal samples. Mice received inoculation of *Salmonella* Heidelberg and then *Escherichia coli* O80:H26 and treatment of no antibiotic (Ctl), ampicillin (Amp), streptomycin (Str) or streptomycin followed by ampicillin (Str–Amp), *n*=6 per treatment group. (**c**) Correlogram shows Pearson’s correlations (the *r* value) between bacterial taxa (phylum and family) and the bacterial amount and gene ratio in the faecal samples. Stars indicate significance correlations (*P<0.05, **P<0.01, ***P<0.001, ****P<0.0001).

In order to determine correlations between gut microbiota and transmission of the *β*-lactam resistance genes, the taxonomic composition of gut microbial communities of mice co-infected with the donor and recipient bacteria was further analysed using 16S rRNA gene amplicon sequencing. In the control group, the composition of gut microbiota was relatively stable. The microbial community was dominated by *Firmicutes *, mainly the families *Ruminoccoccaceae* and *Lachnospiraceae *, and *Bacteroidetes *, mainly the families *Lactobacillaceae* and *Bacteroidaceae* (Fig. 5a, b). In the Amp group, the relative abundance of *Proteobacteria *, mainly the family *Enterobacteriaceae *, increased during the treatment from 0 to 7 days p.i., decreased after the cessation of ampicillin treatment and at 42 days p.i. returned to normal, a range that was not significantly (*P*>0.05) different from the control (Tables S3 and S4). In the Str group, the relative abundance of *Proteobacteri* a, mainly the family *Enterobacteriaceae *, increased on 0, 1 and 2 days p.i. and returned to normal at 7 days p.i. In the Str–Amp group, the relative abundance of Proteobacteria, mainly the family *Enterobacteriaceae *, increased from 0 to 7 days p.i. and returned to normal at 42 days p.i. (Fig. 5a, b). Expansion of *Escherichia–Shigella* and *Salmonella* relative abundance contributed to the increase of *Enterobacteriaceae* relative abundance (Fig. S5). The gut microbial diversity was reduced by the streptomycin pre-treatment and/or ampicillin treatment (Fig. S6). After treatment cessation, the gut microbiota gradually returned towards the original balance (Figs S6 and S7). The correlogram shows that the abundance of tranconjugant and *bla_CMY-2_* gene is positively correlated with the relative abundance of *Proteobacteria* and *Enterobacteriaceae* and negatively with *Firmicutes *, which are unlikely to act as recipients (Fig. 5c). The abundance of *E. coli* O80:H26 and *bla_TEM-1B_* gene are negatively correlated with the relative abundance of *Firmicutes *, *Ruminoccoccaceae* and *Lachnospiraceae *, and the abundance of *S.* Heidelberg is also negatively correlated with the relative abundance of *Firmicutes *.

### Inflammation in the mouse gut

**Fig. 6.**
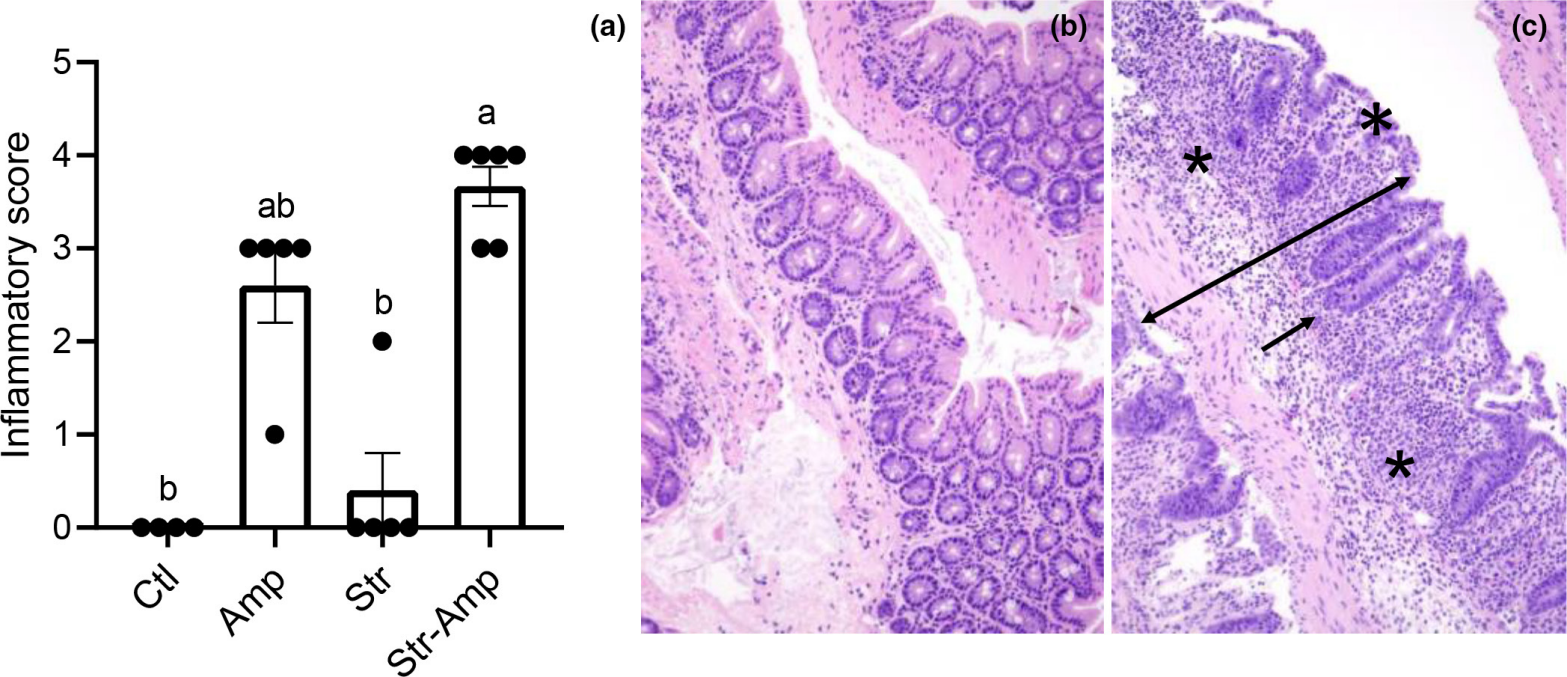
Histological inflammatory scores (mean+se) of the cecum (**a**) in each group of mice that received inoculation of the recipient and donor bacteria and treatment of no antibiotic (Ctl), ampicillin (Amp), streptomycin (Str) or streptomycin followed by ampicillin (Str–Amp), *n*=4, 5, 5 and 6 per group for Ctl, Amp, Str and Str–Amp, respectively. Scores without common letters are of significant (*P*<0.05) difference based on the Kruskal–Wallis test. Representative images of haematoxylin and eosin (H and E)-stained cecum sections: (**b**) normal cecum section with thin mucosa, mature columnar epithelium, regeneration restricted to the crypts, no inflammation in lamina propria and submucosa, and only normal level of mononuclear cells present; (**c**) inflammatory cecum section with transmural inflammation (double-sided arrow), extensive crypt loss (stars), marked crypt hyperplasia (single-sided arrow) and immature cuboidal and attenuated surface epithelium (asterisk).

Cecum specimens were collected at 7 days p.i. from mice co-infected with the *E. coli* O80:H26 donor and *S*. Heidelberg recipient for histopathological analysis. Inflammation was observed in cecum tissue specimens from 100, 20 and 100 % of mice in the Amp, Str and Str–Amp groups, respectively (Fig. 6a). No inflammation was found in the control mice. Fig. 6(b, c) shows the inflamed and normal cecum tissues. The inflammatory score of the Str–Amp group was significantly [H (3)=16.90, *P*<0.001] higher than that of the Str or control groups (Fig. 6a). These scores seemed to be positively associated with the relative abundance of *Proteobacteria* at 7 days p.i. Specifically, the mean inflammatory scores were 3.7, 2.6, 0.4 and 0, and the corresponding mean relative abundance of *Proteobacteria* was 0.8391, 0.4852, 0.0012 and 0.0002 for the Str–Amp, Amp, Str and control groups, respectively (Fig. 5a).

## Discussion

To explore the impact of antibiotics on ARG mobility in pre-disturbed gut microbiota, mice were subjected to streptomycin pre-treatment. The pre-treatment decreased the relative abundance of *Firmicutes *, more specifically *Ruminoccoccaceae* and *Lachnospiraceae *, the short-chain fatty acid producers. Thereby, the treatment would likely have lowered short-chain fatty acid concentrations, increased the luminal pH of the intestine and favoured the colonization of opportunistic pathogens [10, 14], such as the *E. coli* O80:H26 donor and the *S*. Heidelberg recipient. Colonization of the donor and recipient bacteria provided a base for bacterial cell–cell contact in the gut and facilitated conjugative transfer of the *bla_CMY-2_* gene via the IncI2 plasmid in the Str group. In comparison, in the Str–Amp group, despite benefitting the *E. coli* O80:H26 donor in reaching high abundance, ampicillin might kill the actively growing *β*-lactam-susceptible *S*. Heidelberg at the initial stage of infection in the pre-disturbed gut microbiota. Thus, the limited abundance of recipient might lead to a lower conjugation frequency in the Str–Amp group compared to the Str group at 1 day p.i. In support of our findings, Lopatkin *et al.* [19] demonstrated with mathematical models and *in vitro* bacterial culture that antibiotics may reduce conjugation frequency by reducing the sizes of either or both of the parental populations. Hall *et al.* [35] also reported that positive selection for plasmid-encoded traits reduced plasmid conjugation frequency in soil bacterial communities. Here, the abundance of *S*. Heidelberg transconjugants in the Str group increased from 1 to 3 days p.i. and then decreased at 7 days p.i., while that in the Str–Amp group increased from 1 to 7 days p.i. The different dynamics suggest that carrying the IncI2 plasmid to *S*. Heidelberg is a cost in the absence but a benefit in the presence of ampicillin selection pressure. In addition, the relative abundance of commensal *Enterobacteriaceae* expanded significantly during ampicillin treatment. The simultaneous blooming of the donor, transconjugant and commensal *Enterobacteriaceae* in the severely inflamed mouse gut possibly built a strong base for gene transfer among these bacteria. In support of our suggestion, Stecher *et al.* [17] reported that parallel blooms of *S.* Typhimurium and mouse commensal *E. coli* boosted conjugative transfer of a colicin plasmid p2 from S. Typhimurium to *E. coli *. In our study, the *bla_CMY-2_* gene was transferred via the IncI2 plasmid to a mouse commensal *E. coli* O2:H6 strain and incorporated into two locations of its chromosome (Fig. 3), likely through an ISEcp1-mediated transposition [36]. According to the study by Hall *et al.* [35], such physical movement and duplication of genes between plasmid and chromosome is a common way for bacteria to acquire antibiotic resistance. Furthermore, identical IncI1 plasmids carrying no ARGs were found in both the *E. coli* O2:H6 and *S.* Heidelberg transconjugants, suggesting possible transfer of the plasmid from *E. coli* O2:H6 or other commensal bacteria to *S.* Heidelberg. Supporting our findings on complex conjugation among bacteria in the Str–Amp group, Conlan *et al.* [37] reported the dissemination of the carbapenemase gene to multiple bacterial species in a patient during transplant-associated multi-course antibiotic therapies. In the present study, in pre-existing normal microbiota, ampicillin treatment facilitated the co-infection of and conjugation between the donor and recipient. However, the abundance of the donor, recipient and transconjugant were much lower in the Amp group compared to those in the Str–Amp group during the entire study, except that at 1 day p.i. *S.* Heidelberg abundance was slightly higher in the Amp group than the Str–Amp group. The ampicillin treatment alone without bacterial infection could disturb pre-existing normal gut microbiota and reduce *Firmicutes* relative abundance, as shown in our previous study [21]. However, compared to the Str–Amp group, the disturbance in the Amp group was smaller, and the less disturbed microbiota might provide greater colonization resistance to the introduced bacteria and limit their growth at lower abundance. Hence, more *S.* Heidelberg might become dormant at greater colonization resistance in the Amp than Str–Amp group on 1 day p.i., and survive the ampicillin treatment under the protection of *β*-lactamase producing donor [38, 39]. Yet, low abundance of the donor limited the conjugation frequency in the Amp group. In addition, the donor might depend on co-infection with the recipient in establishing colonization herein, as the donor alone failed to colonize the mouse gut under ampicillin treatment in our previous study [21]. Following cessation of ampicillin treatment or removal of selection pressure, the gut microbiota gradually recovered and diminished the introduced bacteria. The more disturbed gut microbiota likely favoured longer persistence of the introduced bacteria in the Str–Amp group compared to other groups. Moreover, under the Str–Amp treatment *S*. Heidelberg reached significantly higher abundance and persisted much longer in mouse faeces following co-infection with *E. coli* O80:H26 compared to *S*. Heidelberg mono-infection. The findings suggest that antibiotic-susceptible opportunistic pathogens may exploit conjugative transfer of ARGs to propagate and persist in an otherwise hostile environment. Overall, pre-disturbed gut microbiota might promote high-abundance colonization of resistant bacteria under positive antibiotic selection pressure and encourage bacterial conjugation and spread of ARGs.

The *E. coli* O80:H26 donor carries one copy of the *bla_CMY-2_* gene and one copy of the *bla_TEM-1B_* gene. The abundance dynamics of the *bla_TEM-1B_* gene and the donor was highly correlated, suggesting that the *bla_TEM-1B_* gene might be transmitted clonally along with the donor. Supporting this suggestion, transconjugant that carries the *bla_TEM-1B_* gene was not recovered in this and previous studies [21]. Using the ratio of *bla_CMY-2_* to *bla_TEM-1B_* as an indicator for dissemination of the *bla_CMY-2_* gene, the significantly higher ratio of the two genes suggested more efficient dissemination of the *bla_CMY-2_* gene in the Str–Amp group than in the Amp group during the entire study, except 1 day p.i. The high ratios might be attributed to the transfer of the *bla_CMY-2_* gene to *S*. Heidelberg and commensal *E. coli *, the incorporation of the *bla_CMY-2_* gene into the commensal *E. coli* chromosome and possible dissemination of the *bla_CMY-2_* gene in the gut microbiota. Furthermore, following cessation of the ampicillin treatment, the abundance of the *bla_CMY-2_* gene remained high, even though the abundance of the donor and transconjugant decreased significantly in the Str–Amp group, suggesting possible persistence of the *bla_CMY-2_* gene in the gut microbiota at no selective advantage.

## Conclusion

In this study, pre-disturbed gut microbiota promoted conjugative transfer of the *bla_CMY-2_* gene from the *E. coli* O80:H26 donor to *S.* Heidelberg, commensal *E. coli* and possibly other commensal *Enterobacteriaceae* under positive ampicillin selection pressure. Following cessation of ampicillin treatment, shedding of the *S.* Heidelberg and *E. coli* transconjugants persisted over 35 days. These findings underline the importance of pre-existing gut microbiota on dissemination of ARGs during antibiotic treatments of bacterial infection.

## Supplementary Data

Supplementary material 1
