# Supplementary material for: Mobility of β-lactam resistance under ampicillin treatment in gut microbiota suffering from pre-disturbance
Source: Microb Genom. 2021 Dec 9;7(12):000713. doi: 10.1099/mgen.0.000713 (PMC8767350; doi:10.1099/mgen.0.000713)
Supplement: Supplementary material 1 [file mgen-7-0713-s001.pdf]

Table S1. Transconjugant isolates

Group	dpi	Genus	Species	Serovar	IncI1	IncI2	*bla*$_{CMY-2}$ location		Accession
							Chromosome	IncI2	(SAMN number)
Amp	7	*Salmonella*	*enterica*	Heidelberg		x		x	16634224
Str	1	*Salmonella*	*enterica*	Heidelberg		x		x	16634202 - 16634207
Str	2	*Salmonella*	*enterica*	Heidelberg		x		x	16634215, 16634216
Str	3	*Salmonella*	*enterica*	Heidelberg		x		x	16634218, 16634219, 16634221
Str	3	*Salmonella*	*enterica*	Heidelberg	x	x		x	16634220
Str	7	*Salmonella*	*enterica*	Heidelberg		x		x	16634225
Str	7	*Salmonella*	*enterica*	Heidelberg	x	x		x	16634226
Str	14	*Salmonella*	*enterica*	Heidelberg	x	x		x	16634231, 16634232
Str-Amp	1	*Salmonella*	*enterica*	Heidelberg		x		x	16634208 - 16634212
Str-Amp	2	*Salmonella*	*enterica*	Heidelberg		x		x	16634217
Str-Amp	3	*Salmonella*	*enterica*	Heidelberg		x		x	16634222, 16634223
Str-Amp	7	*Salmonella*	*enterica*	Heidelberg	x	x		x	16634227, 16634228,
Str-Amp	14	*Escherichia*	*coli*	O2:H6	x	x	x	x	16634233, 16634234, 16634239, 16634240
Str-Amp	14	*Salmonella*	*enterica*	Heidelberg	x	x		x	16634235 - 16634238
Str-Amp	21	*Salmonella*	*enterica*	Heidelberg	x	x		x	16634241 - 16634245
Str-Amp	42	*Salmonella*	*enterica*	Heidelberg	x	x		x	16634246, 16634249, 16634250
Str-Amp	42	*Escherichia*	*coli*	O2:H6	x	x	x	x	16634247, 16634248

Amp = ampicillin treatment, Str = streptomycin treatment, Str-Amp = streptomycin followed by ampicillin treatment, dpi = day post infection

Table S2. Information concerning qPCRs

Gene	Primer sequence (F = forward, R = reverse)	Annealing temperature (°C)	Product length (bp)	Reference
bla_{CMY-2}	F: CAGCATCTCCCAGCCTAATC<br>R: GAAGCCCGTACACGTTTCTC	60	205	Boyer and Singer (2012)
bla_{TEM-1B}	F: CATTTTCGTGTCGCCCTTAT<br>R: GGGCGAAAACTCTCAAGGAT	58	167	Resende. et al. (2014)
16S rRNA	341F: CCTACGGGAGGCAGCAG<br>515R: ATTCCGCGGCTGGCA	60	174	López-Gutiérrez et al. (2004)

Table S3. Significant difference in mouse gut microbial composition at the phylum level*

Sampling day	Bacteroidetes				Firmicutes				Proteobacteria			
	Ctl	Amp	Str	S-A	Ctl	Amp	Str	S-A	Ctl	Amp	Str	S-A
Baseline	a	a	a	a	a	a	a	a	a	a	a	a
0 dpi	a	a	b	b	a	a	b	b	a	a	b	b
1 dpi	a	b	bc	c	a	b	bc	c	a	ab	bc	c
2 dpi	a	b	ab	b	a	b	b	c	a	ab	b	c
7 dpi	a	a	a	b	a	b	a	b	a	b	a	c
14 dpi	a	b	a	b	a	a	a	a	a	b	a	b
21 dpi	a	a	a	b	a	a	a	a	a	b	a	b
42 dpi	a	a	a	a	a	a	a	a	a	a	a	a

*Letters are indicators of difference linked with data in figure 5A. No common letters represent significant ($P < 0.05$) difference in relative abundance of each bacterial phylum on each sampling day among the four treatment groups as determined by Brown-Forsythe and Welch's ANOVA tests, Baseline = 3 days before bacterial inoculation, dpi = day post infection, Ctl = control, Amp = ampicillin, Str = Streptomycin, S-A = Streptomycin followed by ampicillin

Table S4. Significance difference in mouse gut microbial composition at the family level*

Sampling day	Bacteroidaceae				Lactobacillaceae				Lachnospiraceae				Ruminococcaceae				Enterobacteriaceae			
	C	A	S	S-A	C	A	S	S-A	C	A	S	S-A	C	A	S	S-A	C	A	S	S-A
Baseline	a	a	a	a	a	a	a	a	a	a	a	a	a	a	a	a	a	a	a	a
0 dpi	a	a	b	b	a	a	ab	b	a	a	b	b	a	a	b	b	a	a	b	b
1 dpi	a	c	b	c	a	a	b	a	a	b	a	b	a	b	b	b	a	ab	bc	c
2 dpi	a	b	a	b	a	a	b	a	a	b	b	b	a	b	b	b	a	ab	b	c
7 dpi	a	a	a	b	a	a	b	a	a	b	a	b	a	b	b	b	a	b	a	c
14 dpi	a	b	a	b	a	a	a	a	a	a	a	b	a	b	b	b	a	b	a	b
21 dpi	a	b	ab	ab	a	b	a	a	a	a	a	a	a	a	a	b	a	b	a	b
42 dpi	a	a	a	a	a	ab	b	ab	a	a	a	a	a	ab	b	b	a	a	a	a

*Letters are indicators of difference linked with data in figure 5B. No common letters represent significant ($P < 0.05$) difference in relative abundance of each bacterial family on each sampling day among the four treatment groups as determined by Brown-Forsythe and Welch's ANOVA tests, Baseline = 3 days before bacterial inoculation, dpi = day post infection, C = control, A = ampicillin, S = Streptomycin, S-A = Streptomycin followed by ampicillin

Supplemental figure legends

Figure S1. Enumeration of *Escherichia coli* O80:H26, *Salmonella* Heidelberg and the *S.* Heidelberg transconjugant (mean + SE) in fecal samples from mice that received both the recipient and donor inoculation and the treatment of no antibiotic (Ctl), ampicillin (Amp), streptomycin (Str) or streptomycin followed by ampicillin (Str-Amp); n = 10, 11, 11 and 12 for Ctl, Amp, Str and Str-Amp, respectively, by 7 day post infection, and n = 6 per treatment group thereafter; and mean values without common letters are of significant ($P < 0.05$) difference on each sampling day based on Brown-Forsythe and Welch's ANOVA tests.

Figure S2. Enumeration of *S.* Heidelberg (mean + SE) in fecal samples from mice that received both *S.* Heidelberg and *E. coli* O80:H26 inoculation (co-infection) or *S.* Heidelberg inoculation only (mono-infection) under the treatment of no antibiotic [A) Ctl], ampicillin [B) Amp], streptomycin [C) Str] or streptomycin followed by ampicillin [D) Str-Amp]; n = 6 per treatment group, using mice maintained until 42 days post infection; significant (* $P < 0.05$, ** $P < 0.01$, *** $P < 0.001$) difference on each sampling determined by Welch's t-tests

Figure S3. Map of an IncI1 plasmid found in *S.* Heidelberg and *E.coli* O2:H6 transconjugants, rendered with UniPro UGENE v36.0 (http://ugene.net/) and annotated with Prokka: rapid prokaryotic genome annotation v1.13.3 (https://github.com/tseemann/prokka)

Figure S4. Pearson correlation ($P < 0.001$) between the quantity of $bla_{TEM\text{-}1B}$ (A) or $bla_{CMY\text{-}2}$ (B) genes and the number of *E. coli* O80:H26 in individual mouse fecal samples from all antibiotic treatment groups with best fit line (red or blue, solid) and 95% confidence bands (read or blue, dotted); black dash lines represent $y = x$.

Figure S5. Mean relative abundance of Enterobacteriaceae, consisting of *Escherichia-Shigella*, *Salmonella* and other Enterobacteriaceae, based on sequencing of the 16S rRNA gene from mouse fecal samples. Mice received inoculation of *S.* Heidelberg and then *E. coli* O80:H26 and

treatment of no antibiotic (Ctl), ampicillin (Amp), streptomycin (Str) or streptomycin followed by ampicillin (Str-Amp), n = 6 per treatment group, dpi = day post infection.

Figure S6. Microbial richness and evenness as assessed by the Chao1, Shannon and Simpson indexes on each sampling date from various treatment groups: Ctl = control, Amp = ampicillin, Str = streptomycin, Str-Amp = streptomycin followed by ampicillin

Figure S7. Principal coordinate analysis (PCoA) of weighted Bray-Curtis dissimilarity of mouse gut microbiota derived from individual mice on each sampling date from various treatment groups: Ctl = control, Amp = ampicillin, Str = streptomycin, Str-Amp = streptomycin followed by ampicillin

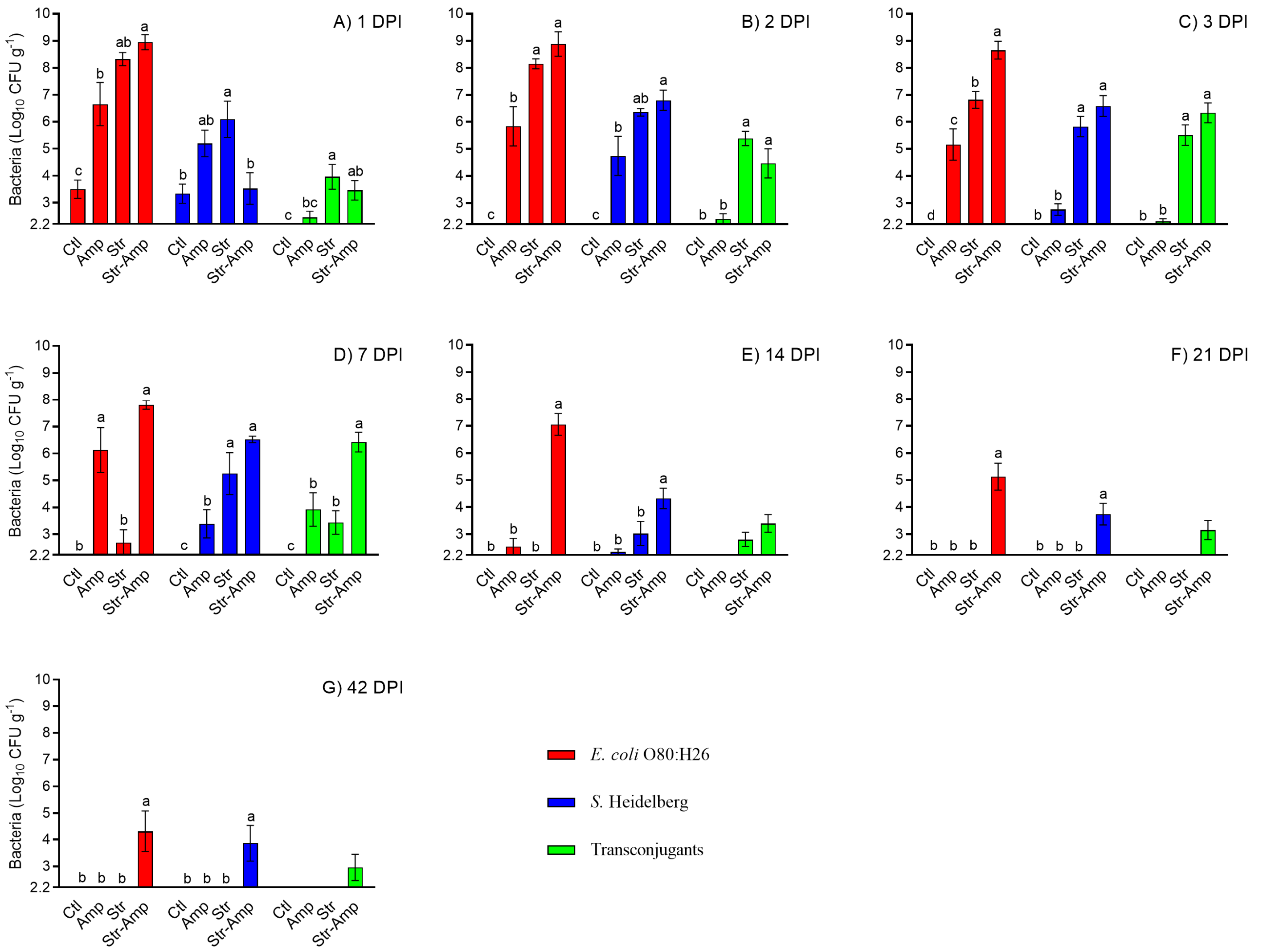
A) 1 DPI
B) 2 DPI
C) 3 DPI
D) 7 DPI
E) 14 DPI
F) 21 DPI
G) 42 DPI
Bacteria (Log10 CFU g-1)
Ctl
Amp
Str
Str-Amp
E. coli O80:H26
S. Heidelberg
Transconjugants

Fig. S1

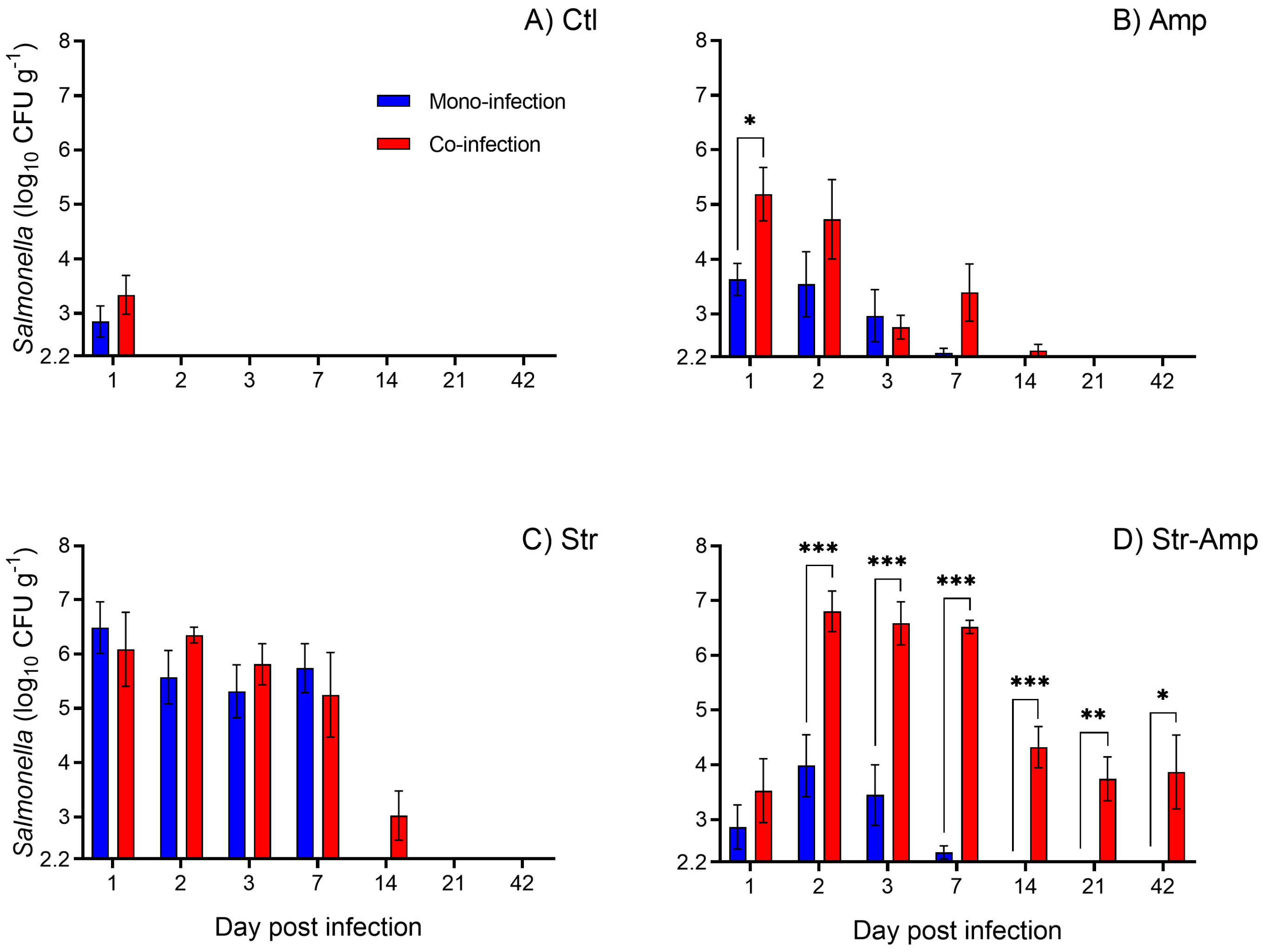
A) Ctl
B) Amp
C) Str
D) Str-Amp
Mono-infection
Co-infection
Salmonella (log10 CFU g-1)
Day post infection
8
7
6
5
4
3
2.2
1
2
3
7
14
21
42
*
**
***

Fig. S2

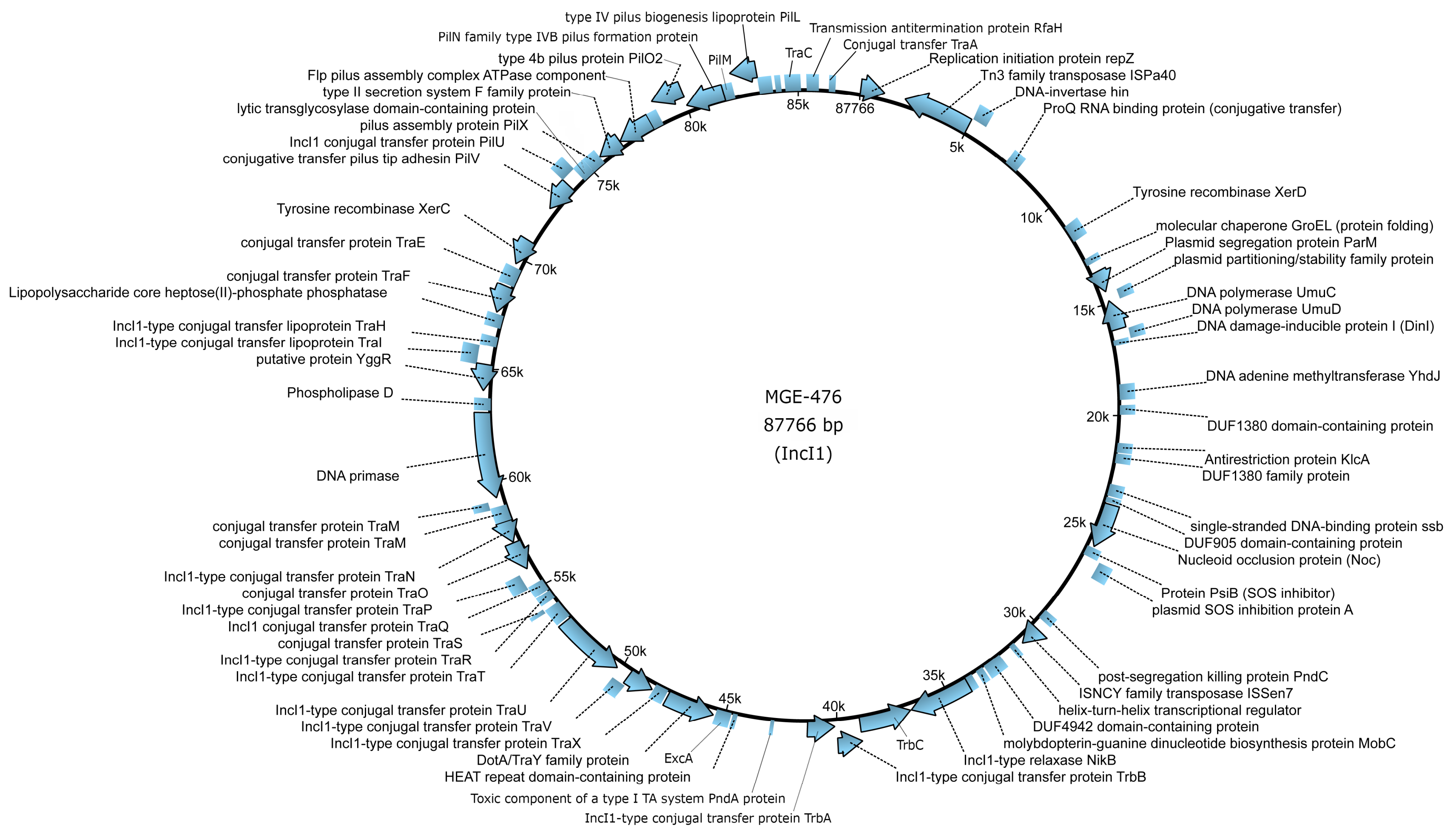
MGE-476
87766 bp
(IncI1)
type IV pilus biogenesis lipoprotein PilL
Transmission antitermination protein RfaH
PilN family type IVB pilus formation protein
TraC
Conjugal transfer TraA
PilM
type 4b pilus protein PilO2
Replication initiation protein repZ
Flp pilus assembly complex ATPase component
Tn3 family transposase ISPa40
type II secretion system F family protein
DNA-invertase hin
lytic transglycosylase domain-containing protein
ProQ RNA binding protein (conjugative transfer)
pilus assembly protein PilX
IncI1 conjugal transfer protein PilU
conjugative transfer pilus tip adhesin PilV
Tyrosine recombinase XerD
Tyrosine recombinase XerC
molecular chaperone GroEL (protein folding)
Plasmid segregation protein ParM
conjugal transfer protein TraE
plasmid partitioning/stability family protein
conjugal transfer protein TraF
Lipopolysaccharide core heptose(II)-phosphate phosphatase
DNA polymerase UmuC
DNA polymerase UmuD
IncI1-type conjugal transfer lipoprotein TraH
DNA damage-inducible protein I (DinI)
IncI1-type conjugal transfer lipoprotein TraI
putative protein YggR
DNA adenine methyltransferase YhdJ
Phospholipase D
DUF1380 domain-containing protein
Antirestriction protein KlcA
DNA primase
DUF1380 family protein
conjugal transfer protein TraM
single-stranded DNA-binding protein ssb
conjugal transfer protein TraM
DUF905 domain-containing protein
Nucleoid occlusion protein (Noc)
IncI1-type conjugal transfer protein TraN
conjugal transfer protein TraO
Protein PsiB (SOS inhibitor)
IncI1-type conjugal transfer protein TraP
plasmid SOS inhibition protein A
IncI1 conjugal transfer protein TraQ
conjugal transfer protein TraS
IncI1-type conjugal transfer protein TraR
IncI1-type conjugal transfer protein TraT
post-segregation killing protein PndC
ISNCY family transposase ISSen7
IncI1-type conjugal transfer protein TraU
helix-turn-helix transcriptional regulator
IncI1-type conjugal transfer protein TraV
DUF4942 domain-containing protein
IncI1-type conjugal transfer protein TraX
molybdopterin-guanine dinucleotide biosynthesis protein MobC
TrbC
DotA/TraY family protein
ExcA
IncI1-type relaxase NikB
HEAT repeat domain-containing protein
IncI1-type conjugal transfer protein TrbB
Toxic component of a type I TA system PndA protein
IncI1-type conjugal transfer protein TrbA

Fig. S3

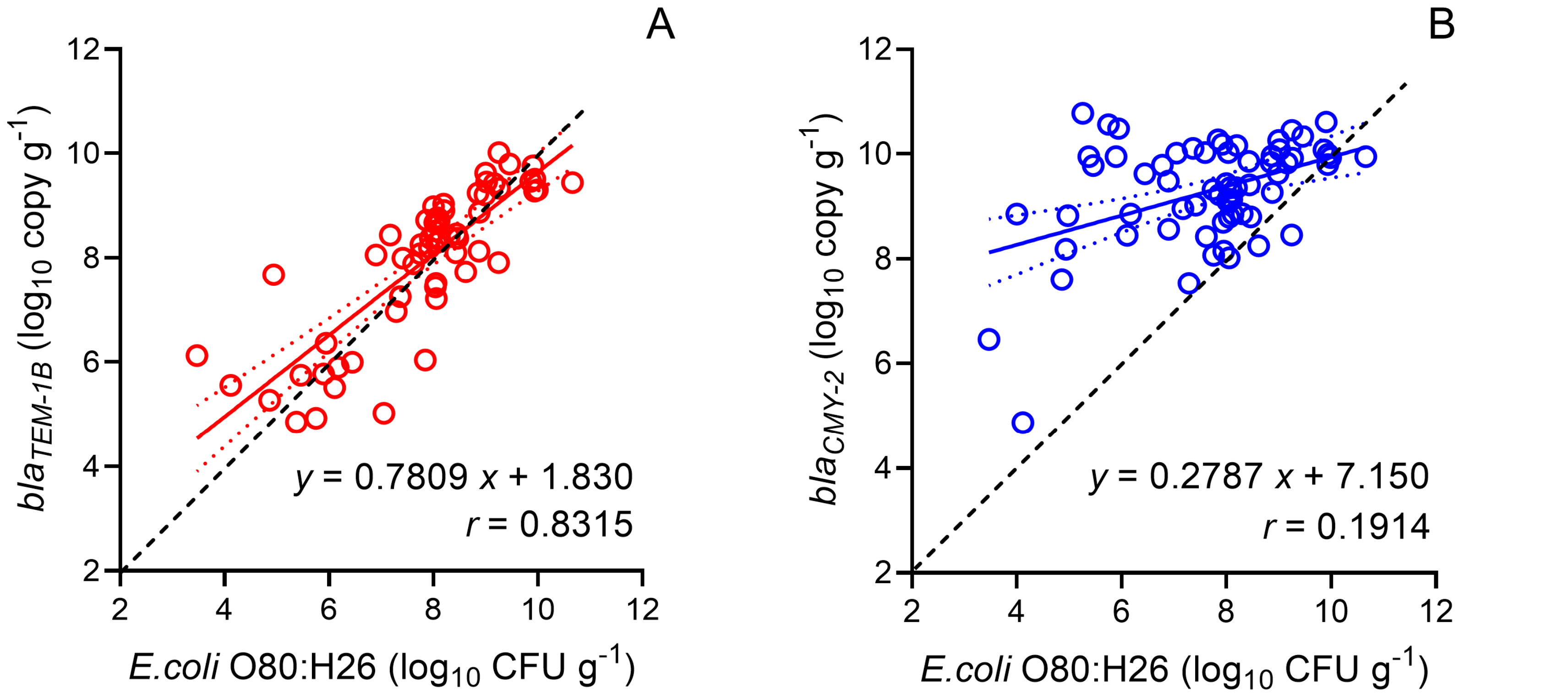
A
B
bla_{TEM-1B} ($\log_{10}$ copy g^{-1})
bla_{CMY-2} ($\log_{10}$ copy g^{-1})
$y = 0.7809\,x + 1.830$
$r = 0.8315$
$y = 0.2787\,x + 7.150$
$r = 0.1914$
E.coli O80:H26 ($\log_{10}$ CFU g^{-1})
E.coli O80:H26 ($\log_{10}$ CFU g^{-1})
2
4
6
8
10
12

Fig. S4

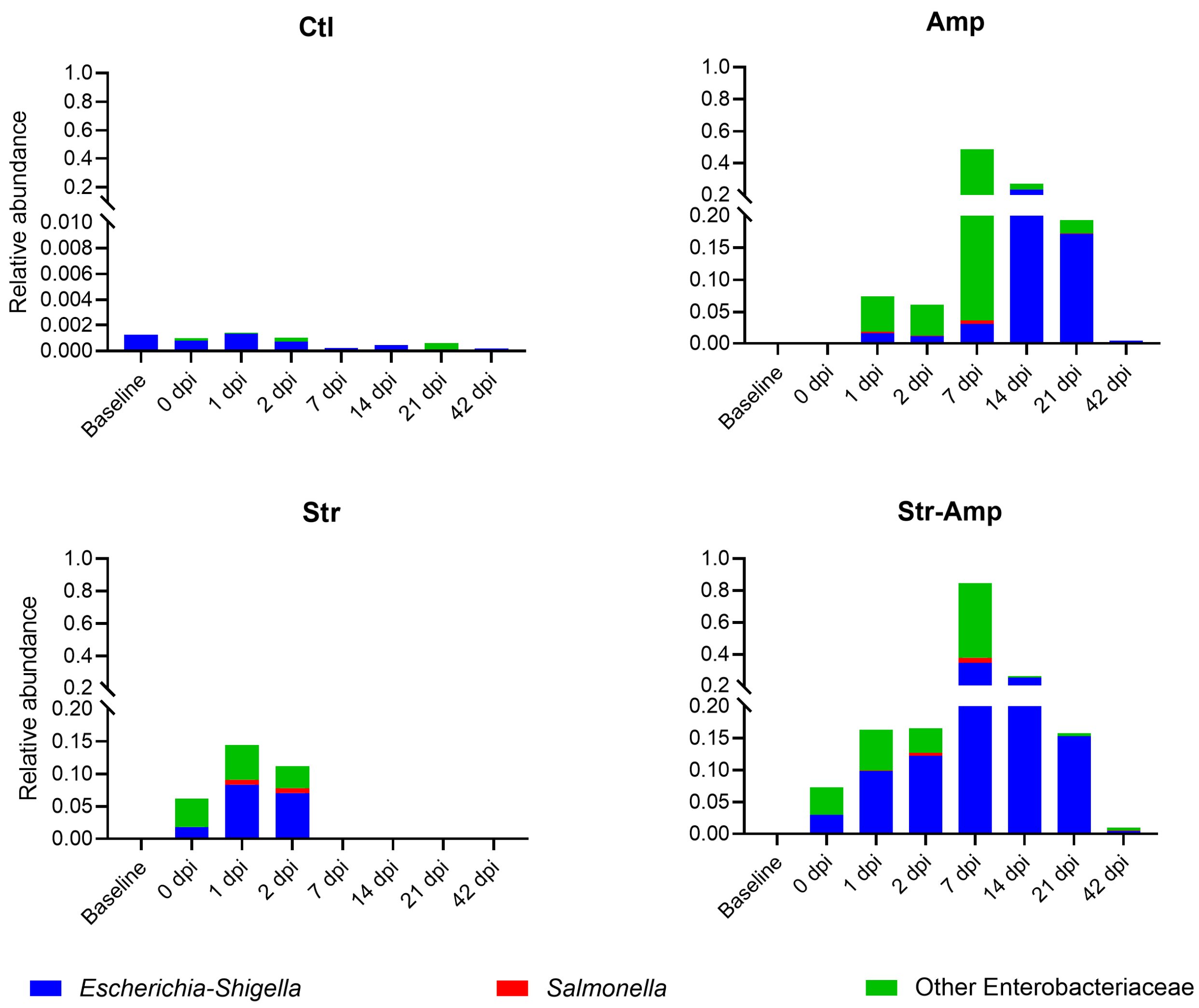
Ctl
Amp
Str
Str-Amp
Relative abundance
1.0
0.8
0.6
0.4
0.2
0.010
0.008
0.006
0.004
0.002
0.000
0.20
0.15
0.10
0.05
0.00
Baseline
0 dpi
1 dpi
2 dpi
7 dpi
14 dpi
21 dpi
42 dpi
Escherichia-Shigella
Salmonella
Other Enterobacteriaceae

Fig. S5

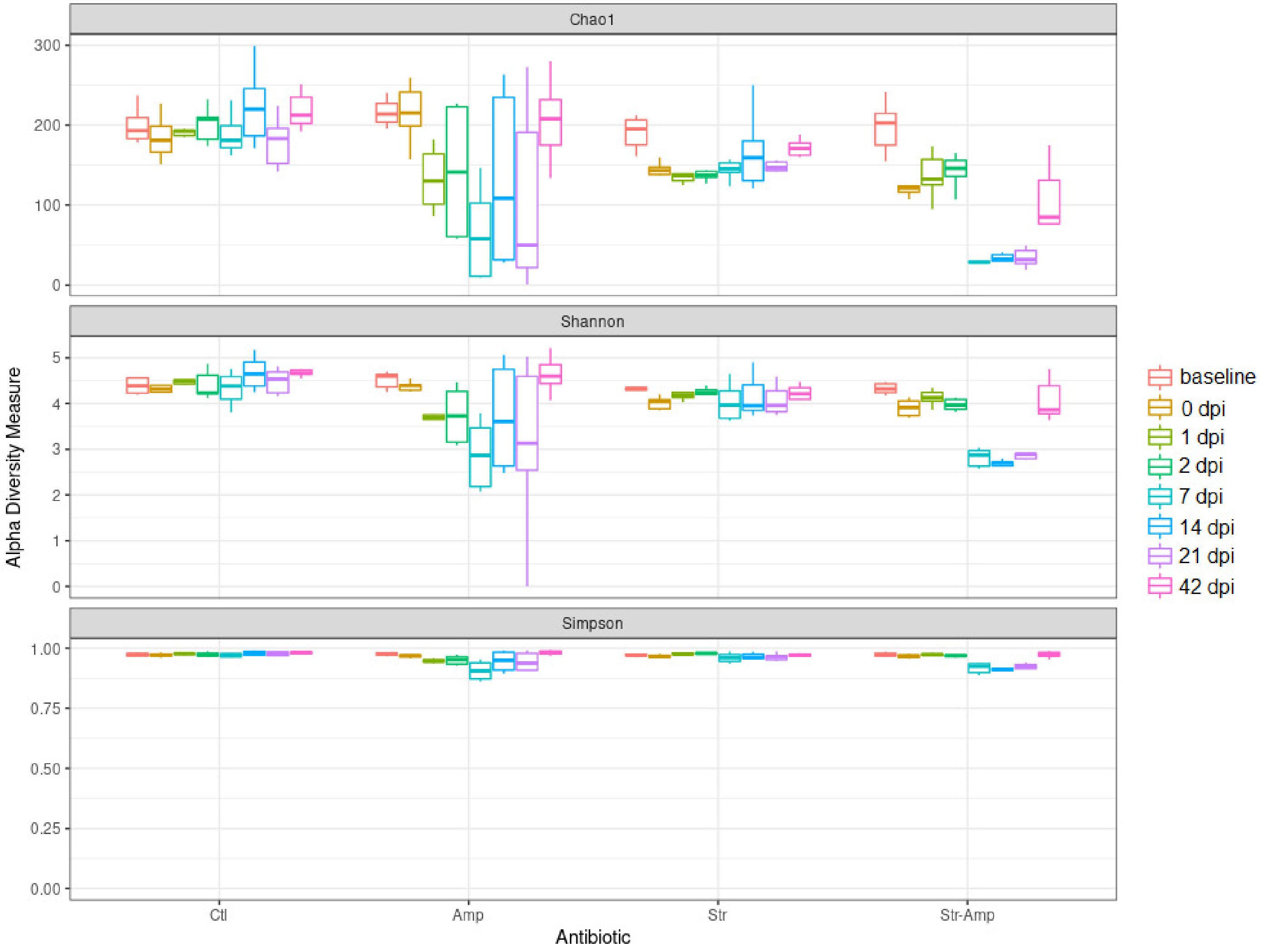
Chao1
Shannon
Simpson
Alpha Diversity Measure
300
200
100
0
5
4
3
2
1
0
1.00
0.75
0.50
0.25
0.00
Ctl
Amp
Str
Str-Amp
Antibiotic
baseline
0 dpi
1 dpi
2 dpi
7 dpi
14 dpi
21 dpi
42 dpi

Fig. S6

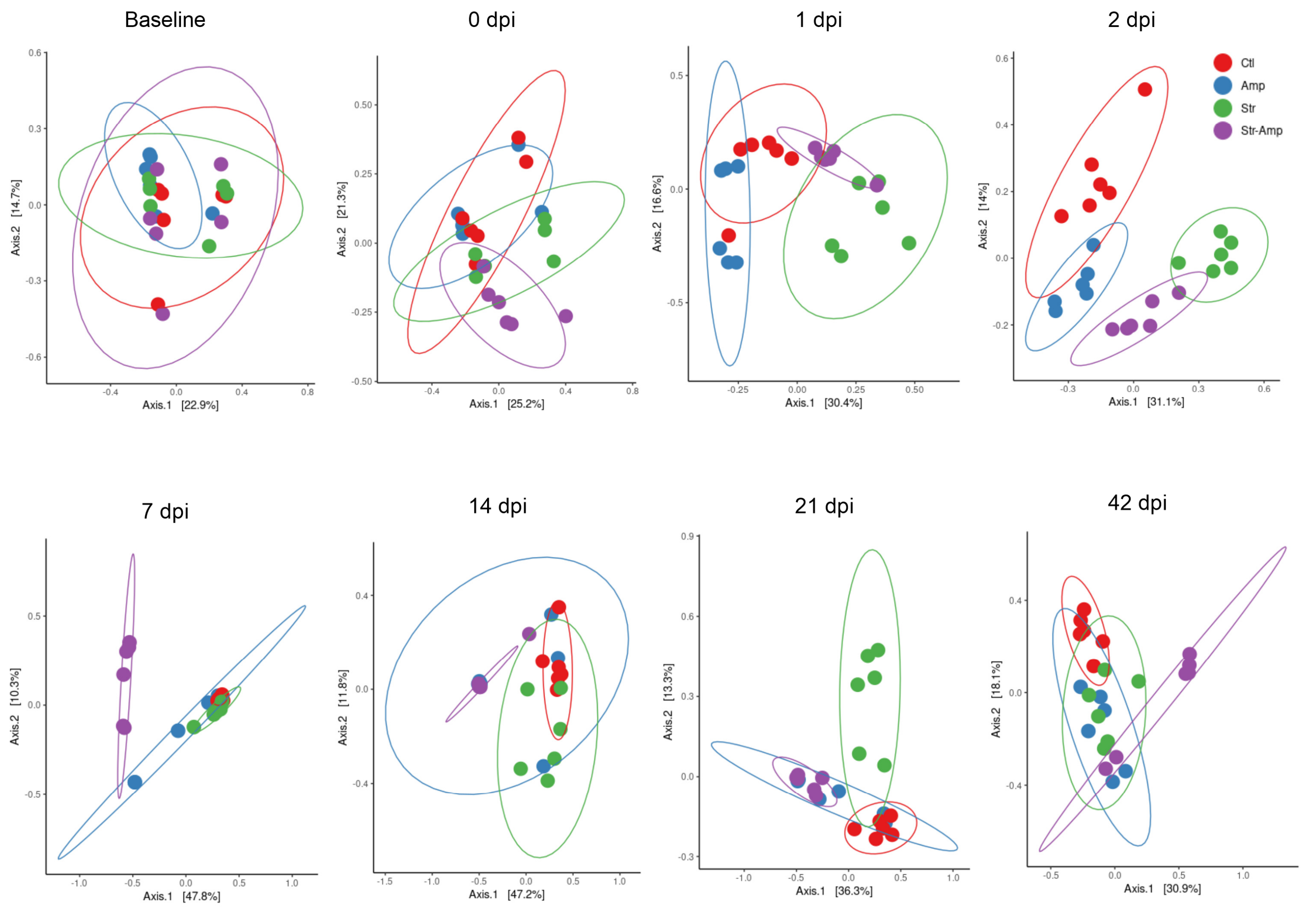
Baseline
0 dpi
1 dpi
2 dpi
7 dpi
14 dpi
21 dpi
42 dpi
Ctl
Amp
Str
Str-Amp
Axis.1 [22.9%]
Axis.2 [14.7%]
Axis.1 [25.2%]
Axis.2 [21.3%]
Axis.1 [30.4%]
Axis.2 [16.6%]
Axis.1 [31.1%]
Axis.2 [14%]
Axis.1 [47.8%]
Axis.2 [10.3%]
Axis.1 [47.2%]
Axis.2 [11.8%]
Axis.1 [36.3%]
Axis.2 [13.3%]
Axis.1 [30.9%]
Axis.2 [18.1%]

Fig. S7
